# Hierarchical reorganization of cortical and cerebellar functional gradients in Parkinson’s disease

**DOI:** 10.3389/fneur.2026.1942024

**Published:** 2026-10-06

**Authors:** Qiang Liu, Zezhong Zheng, Moxuan Zhang, Siyu Zhou, Qichao Cheng, Luhao Cui, Shanshan Mei, Ruihua Hou, Teng Chen, Yilei Xiao, Fangang Meng

**Affiliations:** 1 Beijing Neurosurgical Institute, Beijing Tiantan Hospital, Capital Medical University, Beijing, China; 2 Department of Neurosurgery, Shandong First Medical University Cancer Hospital, Jinan, China; 3 Department of Radiology, Qilu Hospital of Shandong University, Jinan, China; 4 Department of Neurology, Xuanwu Hospital, Capital Medical University, Beijing, China; 5 Department of Psychiatry, Clinical and Experimental Sciences, Faculty of Medicine, University of Southampton, Southampton, United Kingdom; 6 Department of Neurosurgery, Qilu Hospital of Shandong University, Jinan, China; 7 Department of Neurosurgery, Liaocheng People's Hospital, Liaocheng, China

**Keywords:** essential tremor, functional connectivity, functional gradient, machine learning, Parkinson ‘s disease, rest-state fMRI

## Abstract

**Introduction:**

Parkinson’s disease (PD) is a common movement disorder with heterogeneous clinical manifestations, and its tremor-predominant presentations may overlap with essential tremor (ET), complicating clinical differentiation in some patients. Functional gradient analysis provides a continuous framework for characterizing macroscale brain hierarchical organization and may offer candidate imaging features for disease characterization.

**Methods:**

We first investigated surface-based resting-state functional measures and cerebral perfusion in 50 patients with idiopathic PD and 33 healthy controls. We subsequently incorporated functional MRI data from 17 patients with ET to examine alterations in cortical and cerebellar functional gradients in PD and ET and to evaluate their potential utility for distinguishing the two disorders. Group comparisons, gradient range and variance analyses, network-level analyses, and multiclass support vector machine classification were performed.

**Results:**

Patients with PD showed widespread abnormalities in both cortical and cerebellar Gradient 1 and Gradient 2, whereas patients with ET exhibited fewer cortical alterations and no significant cerebellar gradient abnormalities. At the group level, both PD and ET showed broader cortical gradient ranges; however, only PD demonstrated significantly increased individual-level gradient range and variance. Network-level analyses further revealed pronounced hierarchical redistribution between sensorimotor and higher-order associative systems in PD, while ET displayed relatively limited alterations. The combined use of cortical and cerebellar Gradient 1 and Gradient 2 features showed discriminatory performance within the present dataset, with area under the receiver operating characteristic curve values of 1.000 for healthy controls, 0.960 for PD, and 0.889 for ET. Shapley additive explanations (SHAP) analysis indicated that both cerebral and cerebellar gradient features contributed to classification, with broader feature involvement in PD than in ET.

**Discussion:**

These findings suggest that PD is characterized by extensive disruption of cortical-cerebellar functional hierarchy, exceeding the relatively limited alterations observed in ET. Functional gradients may therefore provide candidate imaging features for characterizing PD-related hierarchical reorganization and for exploratory differentiation of PD from ET.

## Introduction

Parkinson’s disease (PD) and essential tremor (ET) are clinically distinct movement disorders, but tremor-predominant presentations may overlap and complicate differential diagnosis in some patients. Although advanced ET does not directly evolve into PD, growing evidence suggests a close association between the two conditions (1). In particular, patients with ET appear to have an increased risk of developing PD (2), and a subset may exhibit PD-like features during the disease course, a condition often described as essential tremor-Parkinson’s disease syndrome (ET-PD) (3, 4). This clinical overlap highlights the importance of accurate differentiation between the two disorders. At present, however, differentiation still relies largely on characteristic symptom evaluation and pharmacological testing, while the distinction between ET-PD and PD remains insufficiently objective (5). This diagnostic uncertainty highlights the need to identify clinical and neuroimaging indicators that may improve the characterization and differential assessment of these disorders.

In recent years, with the continuous development of novel magnetic resonance imaging techniques, functional magnetic resonance imaging (fMRI) has assumed an increasingly important role in the characterization and differential diagnosis of neurodegenerative diseases (6). Unlike conventional structural MRI, which primarily reflects relatively late morphological alterations such as brain atrophy, fMRI measures blood oxygen level-dependent (BOLD) signals and is capable of revealing abnormalities in functional connectivity (FC) and the dynamic reorganization of large-scale brain networks (7). As such, it provides a means of detecting network-level dysfunction at relatively early stages of disease. In PD, resting-state fMRI studies have demonstrated abnormal functional connectivity within the basal ganglia-thalamo-cortical circuit, as well as within motor, visual, and executive control networks (8). Some studies have further suggested that functional networks may exhibit compensatory increases in connectivity or pathological hyper-efficiency during the early stages of PD (9). In contrast, in dementia and its prodromal stage, mild cognitive impairment (MCI), reduced functional connectivity within the default mode network (DMN) is considered one of the most robust imaging biomarkers (10). In particular, decreased connectivity between the posterior cingulate cortex and the medial prefrontal cortex has been closely associated with memory impairment (11). In addition, when combined with graph-theoretical analysis, fMRI can quantify the topological properties of whole-brain networks, including small-worldness, global efficiency, and modularity, thereby providing quantitative evidence for disease-specific differences in network organization (12, 13). In the context of differential diagnosis, fMRI may be particularly valuable for distinguishing disorders that share similar clinical manifestations but differ in their underlying pathophysiological mechanisms. For example, abnormalities in visual and attentional networks are more pronounced in dementia with Lewy bodies, whereas frontotemporal dementia is more prominently characterized by disruption of frontotemporal network connectivity (14). Furthermore, through multi-network analyses and machine-learning approaches, such as support vector machines and random forests, functional connectivity patterns can be leveraged to classify different neurodegenerative disorders and improve diagnostic accuracy at the individual level (15–18).

Brain gradients have recently emerged as an important concept in macroscale brain network research, offering a novel framework for characterizing the hierarchical organization of functional and structural connectivity in a continuous spatial manner (19). Unlike traditional parcellation-based approaches, which divide the brain into discrete networks, gradient analysis emphasizes continuous axes of variation across brain regions, such as transitions from lower-order to higher-order processing and from local specialization to integrative function (20). This approach therefore provides insight into the macroscale organizational principles of the brain within topological space. At the level of functional connectivity, dimensionality reduction techniques such as diffusion embedding and Laplacian eigenmaps can project high-dimensional resting-state fMRI connectivity matrices onto a set of principal gradients. Seminal studies have shown that the first principal gradient typically reflects a hierarchical axis extending from primary sensorimotor cortices to trans-modal association regions, such as DMN, thereby capturing the transition from externally oriented sensory processing to internally oriented cognitive processing. This continuous axis provides a unified framework for understanding the hierarchical integration of brain function from perception to abstract cognition (19). In neurodegenerative diseases, gradient analysis offers a novel perspective for elucidating disease-related network reorganization. For example, in Alzheimer’s disease, the transmodal, higher-order end of the gradient, where the DMN is typically located, often shows gradient compression, reflecting disrupted functional differentiation (21, 22). In PD, the gradient distribution between sensorimotor and cognitive-integrative regions may also be altered, suggesting a breakdown of the brain’s functional hierarchy (23, 24). Furthermore, dopaminergic medication has been shown to partially restore abnormal functional-gradient organization, indicating that macroscale hierarchical architecture is sensitive to dopamine-dependent network modulation (25). Accordingly, gradient-based metrics have increasingly been used to quantify the degree of hierarchical network disorganization and have been shown to correlate with cognitive performance, clinical stage, and other disease-related variables.

Previous work has therefore established that functional-gradient organization is altered in PD, including whole-brain hierarchical abnormalities (23), striatal gradient changes associated with cognitive impairment (24), and dopamine-sensitive modulation of functional gradients (25). However, these studies have largely focused on PD relative to healthy controls or on disease-specific alterations within selected neural systems. It remains unclear whether PD and ET, despite partially overlapping tremor manifestations, show distinguishable patterns of hierarchical reorganization across both the cerebral cortex and cerebellum. In addition, the relationship between macroscale hierarchical alterations and complementary measures of spontaneous activity, local synchronization, connectivity density, and cerebral perfusion has not been fully characterized. Addressing these gaps may help determine whether PD is associated with broader cortical-cerebellar hierarchical disruption than ET and clarify how gradient abnormalities relate to conventional functional and physiological changes.

Accordingly, the present study aimed to characterize cortical and cerebellar functional hierarchy in PD and to determine whether its pattern differs from ET. We first examined surface-based amplitude of low-frequency fluctuations (ALFF), regional homogeneity (ReHo), global functional connectivity density (gFCD), and cerebral blood flow (CBF) in PD relative to healthy controls to provide complementary context for regional functional and perfusion abnormalities. Functional gradients, which were derived directly from FC matrices, constituted the principal analysis for the three-group comparison. Specifically, we asked whether (1) PD shows more extensive cortical and cerebellar gradient alterations than ET, (2) the two disorders exhibit distinct network-level redistribution across sensorimotor, attentional, and transmodal systems, and (3) gradient-derived features contain exploratory discriminative information for distinguishing PD, ET, and healthy controls. The ET cohort was therefore included as a clinically overlapping comparison group rather than as evidence for early diagnostic performance.

## Methods

### Subjects and assessments

This study was conducted in accordance with the Declaration of Helsinki and was approved by the Ethics Committee of Beijing Tiantan Hospital. Written informed consent was obtained from all participants prior to all study-related examinations. Between September 2024 and September 2025, a total of 50 patients with idiopathic Parkinson’s disease, 17 patients with essential tremor, and 33 healthy controls were consecutively enrolled. Although the three groups did not differ significantly in sex distribution, PD group was significantly older than the healthy control group. All participants, including patients and healthy controls, were recruited from the outpatient and inpatient services of the Department of Functional Neurosurgery. For all patient groups, clinical assessments were performed after withdrawal of antiparkinsonian or other related medications for at least 12 h, and all evaluations were completed in a single session whenever feasible. All participants underwent a standardized neuropsychological and psychiatric assessment, including the Mini-Mental State Examination (MMSE), Montreal Cognitive Assessment (MoCA), Hamilton Depression Rating Scale (HAMD), and Hamilton Anxiety Rating Scale (HAMA). In addition, patients with PD and ET underwent further disease-related evaluations using the Unified Parkinson’s Disease Rating Scale (UPDRS), Non-Motor Symptoms Scale (NMSS), Fahn-Tolosa-Marín Tremor Rating Scale (FTM-TRS), The Essential Tremor Rating Assessment Scale (TETRAS), and the Quality of Life in Essential Tremor Questionnaire (QUEST). (Table 1.) All clinical rating scales were independently scored by three neurosurgeons, and the final score for each scale was calculated as the mean of the three evaluations.

**Table 1:** Group information and clinical characteristics.

Characteristics	Healthy Subjects (*n* = 33)	PD (*n* = 50)	ET (*n* = 17)	*P* value
Age (years)	51.63 ± 11.64	63.82 ± 6.94	48.82 ± 14.94	*P_1_ <* 0.05 *P_2_* = 0.48
Gender (male/female)	13/20	26/24	10/7	*P ^#^* = 0.26
Disease duration (years)	-	9.65 ± 3.98	6.28 ± 3.93	-
Hoehn-Yahr stage	-	2.65 ± 0.73	-	-
UPDRS Part III	-	52.41 ± 22.35	8.67 ± 5.42	-
NMSS	-	48.65 ± 24.78	7.39 ± 6.42	-
MMSE	28.32 ± 1.41	25.48 ± 1.42	27.48 ± 2.31	*P_1_* < 0.05 *P_2_* = 0.45
MoCA	27.42 ± 2.13	23.61 ± 3.02	26.34 ± 2.56	*P_1_* < 0.05 *P_2_* = 0.12
HAMD	5.84 ± 1.78	16.31 ± 5.04	7.32 ± 2.85	*P_1_* < 0.05 *P_2_* = 0.08
HAMA	4.36 ± 2.94	18.43 ± 3.36	11.45 ± 4.85	*P_1_* < 0.05 *P_2_* < 0.05
FTM-TRS	-	14.54 ± 3.72	30.32 ± 12.14	-
TETRAS	-	-	18.45 ± 9.48	*-*
QUEST	-	-	17.63 ± 8.69	*-*

#Chi-square test; PD, Parkinson’s disease; ET, essential tremor. P_1_, PD versus healthy Subjects; P_2_, ET versus healthy subjects. All data were reported as Mean±SD.

### MRI scanning

All MRI scans were performed at the department of radiology in Beijing Tiantan Hospital, using a 3.0-T MRI scanner (Siemens Prisma). All participants underwent the same imaging protocol with identical acquisition parameters, including T1-weighted magnetization-prepared rapid gradient-echo (MPRAGE), BOLD and arterial spin labeling (ASL) sequences. However, due to poor compliance among patients with ET, the quality of ASL data in this group was generally suboptimal; therefore, ASL-related analyses were not included in the present study. The imaging parameters were as follows. For T1-weighted MPRAGE, the repetition time (TR) was 2,530 ms, the echo time (TE) was 1.94 ms, the inversion time (TI) was 1.1 s, the flip angle was 7°, the slice thickness was 1 mm, the acquisition matrix was 256 × 256, the encoding direction was sagittal, and the number of slices was 192. For BOLD imaging, the TR was 2000 ms, the TE was 30 ms, the slice thickness was 3.2 mm, the number of axial slices was 44, the flip angle was 90°, the field of view (FOV) was 22.0 × 22.0 cm, the acquisition matrix was 64 × 64, the number of time points was 240, and the voxel size was 3.4 × 3.4 × 3.2 mm. For ASL imaging, a three-dimensional pulsed arterial spin labeling (3D-PASL) sequence was acquired with a labeling duration of 700 ms, a post-labeling delay (PLD) of 1.99 s, a TR of 4,000 ms, a TE of 25.24 ms, an acquisition matrix of 64 × 64, six acquisition time points, and a voxel size of 3 × 3 × 3 mm. In addition, two identical gradient-echo (GRE) sequences were acquired for each participant to enable field map correction when substantial geometric distortion was observed on BOLD images. Routine T2-weighted imaging, fluid-attenuated inversion recovery (FLAIR), and diffusion-weighted imaging (DWI) were also performed to exclude coexisting central nervous system abnormalities that might affect subsequent analyses, including ischemic lesions, vascular malformations, and intracranial tumors.

### MRI data processing

All acquired MRI data were organized in accordance with the Brain Imaging Data Structure (BIDS) (26). Functional MRI data were preprocessed using fMRIPrep (27), including structural T1-weighted image registration, tissue segmentation, cortical surface reconstruction, and BOLD preprocessing steps such as slice-timing correction, head-motion correction, and spatial normalization. The normalized BOLD data were subsequently postprocessed using XCP-D in CIFTI format (28). Postprocessing included despiking, linear detrending, nuisance regression using the 36-parameter model, temporal band-pass filtering at 0.01–0.08 Hz, and motion censoring at a framewise displacement threshold of 0.3 mm. The 36-parameter model comprised six rigid-body motion parameters, mean white-matter, cerebrospinal-fluid, and global signals, together with their temporal derivatives, squared terms, and squared derivatives. ALFF was calculated as the mean square root of the power spectrum within 0.01–0.08 Hz, whereas cortical ReHo was quantified using Kendall’s coefficient of concordance between each vertex and its immediately adjacent vertices on the fsLR surface mesh. Functional connectivity was defined as the Pearson correlation coefficient between pairs of denoised vertex-wise BOLD time series. Global functional connectivity density was subsequently calculated from the vertex-wise correlation matrix as the number of positive connections exceeding *r* > 0.60 (29):


$$\text{gFCD}=\sum_{\begin{array}{c} j=1\\ j\neq i \end{array}}^{N}I(r_{\mathrm{ij}}>0.60)$$

Where *r_ij_* denotes the Pearson correlation between vertices *i* and *j*, and *I(·)* is the indicator function. Self-connections were excluded by setting the diagonal elements of the correlation matrix to zero. ALFF and ReHo were calculated before spatial smoothing, and the resulting metric maps were subsequently smoothed using a 6-mm full-width at half-maximum Gaussian kernel. The FC matrices were used as intermediate inputs for the calculation of gFCD and functional gradients rather than as independent imaging outcomes.

In addition, ASL data were preprocessed using ASLPrep (30), including coregistration, spatial normalization, distortion correction, noise component extraction, tissue segmentation, skull stripping, cerebral blood flow (CBF) quantification, CBF denoising, and partial volume correction. Notably, fMRIPrep, XCP-D, and ASLPrep all follow the HCP (Human Connectome Project) fsLR standard space for cortical spatial normalization (31). Accordingly, all cerebral cortical metrics reported in this study were computed in fsLR space, whereas metrics related to the cerebellum and subcortical structures were calculated in Montreal Neurological Institute (MNI) space (32). Further implementation details and the corresponding analysis code are provided in the Supplementary material.

### Brain gradient computation

For the preprocessed cortical FC networks, individual gradient maps were constructed for each participant using the BrainSpace toolbox (33). A similarity matrix was generated using the normalized angle kernel, and dimensionality reduction was performed using diffusion map embedding. The resulting individual cortical gradient maps were then aligned to the Schaefer 400-parcel gradient template (34) derived from HCP resting-state fMRI data to facilitate subsequent group-level statistical analyses. Similarly, cerebellar gradient maps were constructed from cerebellar FC networks using the same analytical framework. Individual cerebellar gradients were subsequently aligned to a standard cerebellar gradient template, and gradient values were extracted across different functional networks according to the Yeo network framework (35). Previous studies have generally suggested that the first gradient (Gradient 1) reflects the degree of functional differentiation across brain regions, with values spanning a continuum from primary sensorimotor networks to higher-order transmodal networks. In contrast, the second gradient (Gradient 2) is thought to capture variation across sensory modalities within primary regions and to reflect the intrinsic organization of regional functional activity, with values extending from the visual network to the auditory and somatomotor networks (19). By comparison, the physiological significance of the third and higher-order gradients remains less well understood. Therefore, the present study focused exclusively on the first two gradients, including analyses of regional gradient values, comparisons of gradient range, assessment of gradient standard deviation, and network-level analyses based on the Yeo’s functional networks (36).

## Statistics analysis

Demographic and clinical characteristics were compared among PD, ET, and HC groups. Continuous variables were assessed using the Kruskal-Wallis test, followed by pairwise Mann–Whitney U tests when appropriate, whereas sex distribution was compared using the chi-square test. Variables showing significant between-group differences were considered potential confounders. Group-level analyses of ALFF, ReHo, and gFCD were initially performed between PD and HC because these conventional cortical metrics were included primarily to characterize PD-related functional abnormalities. General linear models were applied at each cortical vertex, with age, sex, mean head motion, HAMD, and HAMA included as nuisance covariates where appropriate. Multiple comparisons were corrected using random field theory, with a vertex-level threshold of 100 mm^2^ and a cluster-level corrected threshold of *P_RFT_* < 0.05. Given the relatively small ET sample, ET-related analyses were considered exploratory and were interpreted cautiously. Because the ASL data acquired in ET group did not meet the predefined quality-control criteria, analyses of CBF and BOLD-CBF coupling were restricted to PD and HC. Within each group, vertex-wise coupling between CBF and ALFF, ReHo, and gFCD was assessed using Pearson correlation analysis, with *p* < 0.05 considered statistically significant. For gradient analyses, two-sample *t*-tests were used to assess regional differences in gradient measures between PD and HC and between ET and HC, with false discovery rate (FDR) correction applied for multiple comparisons. In addition, the variability of cortical gradients in PD and ET groups was compared with that in HC group using *t*-tests. Gradient values across different Yeo’s networks in both the cortex and cerebellum were also compared using *t*-tests. Finally, machine-learning classification models based on whole-brain network gradients were constructed using scikit-learn (37) to distinguish among PD, ET, and HC, with the aim of evaluating the exploratory discriminative value of gradient-derived features rather than establishing a clinically validated diagnostic biomarker.

## Results

### Demographic and clinical characteristics

The demographic and clinical characteristics of the participants are summarized in Table 1. The three groups did not differ significantly in sex distribution (*p* = 0.26). Compared with HC group, patients with PD were significantly older and had significantly lower MMSE and MoCA, together with higher HAMD and HAMA (all *p* < 0.05). The mean disease duration of PD group was 9.65 ± 3.98 years, and the mean Hoehn-Yahr stage was 2.65 ± 0.73, indicating that the cohort predominantly comprised patients with moderate-stage PD. The mean UPDRS Part III and NMSS scores in the PD group were 52.41 ± 22.35 and 48.65 ± 24.78, respectively. Patients with ET did not differ significantly from healthy controls in age, MMSE, MoCA, or HAMD (all *p* > 0.05), but showed significantly higher HAMA (*p* < 0.05). The mean disease duration in the ET group was 6.28 ± 3.93 years. The mean FTM-TRS, TETRAS, and QUEST were 30.32 ± 12.14, 18.45 ± 9.48, and 17.63 ± 8.69, respectively. These clinical differences were considered when interpreting the subsequent imaging findings.

### PD-specific cortical metric analyses

After RFT correction, the results were reported according to the Desikan-Killiany atlas. Compared with healthy controls, patients with PD exhibited predominantly reduced ALFF in regions belonging to DMN, including the right isthmus cingulate cortex and middle temporal gyrus, as well as in frontoparietal control network (FPN), including the left inferior frontal gyrus (pars triangularis) and superior frontal gyrus. Additional ALFF reductions were observed in regions associated with limbic network (LIM) and dorsal attention network (DAN), including the left inferior frontal gyrus, right middle temporal gyrus, right supramarginal gyrus, and left superior temporal gyrus. In contrast, patients with PD showed increased ALFF in regions within visual network (VN) and somatomotor network (SMN), including the right lateral occipital cortex, right precentral gyrus, and left postcentral gyrus, suggesting possible compensatory hyperactivity. Increased ALFF was also observed in several DMN regions. For ReHo, patients with PD demonstrated significantly reduced values in salience network regions, particularly the bilateral insula. By contrast, significantly increased ReHo was identified in the bilateral central regions and the right superior parietal lobule, mainly involving SMN and DAN. With respect to gFCD, patients with PD showed significantly decreased gFCD in visual network regions, including the bilateral fusiform gyrus, left lateral occipital cortex, left inferior temporal gyrus, and right cuneus. Reduced gFCD was also observed in the frontoparietal control network, including the bilateral superior frontal gyrus, left inferior frontal gyrus, left posterior anterior cingulate cortex, and left rostral middle frontal gyrus, as well as in the limbic network, including the bilateral lateral orbitofrontal cortex and right temporal pole. Conversely, significantly increased gFCD was found in DMN regions, such as the bilateral isthmus cingulate cortex and right inferior parietal lobule, as well as in the bilateral central regions within SMN. Finally, cortical perfusion abnormalities were also identified in PD. CBF was significantly reduced in the frontoparietal control network, including the bilateral superior frontal gyrus and right rostral middle frontal gyrus, and in the limbic network, including the right temporal pole and left orbitofrontal cortex. Decreased CBF was also observed in certain DMN regions, such as the left superior temporal gyrus. In contrast, significantly increased CBF was detected in the bilateral central regions of SMN, while elevated CBF was also found in DAN-related regions, including the right supramarginal gyrus (Figure 1).

**Figure 1:**
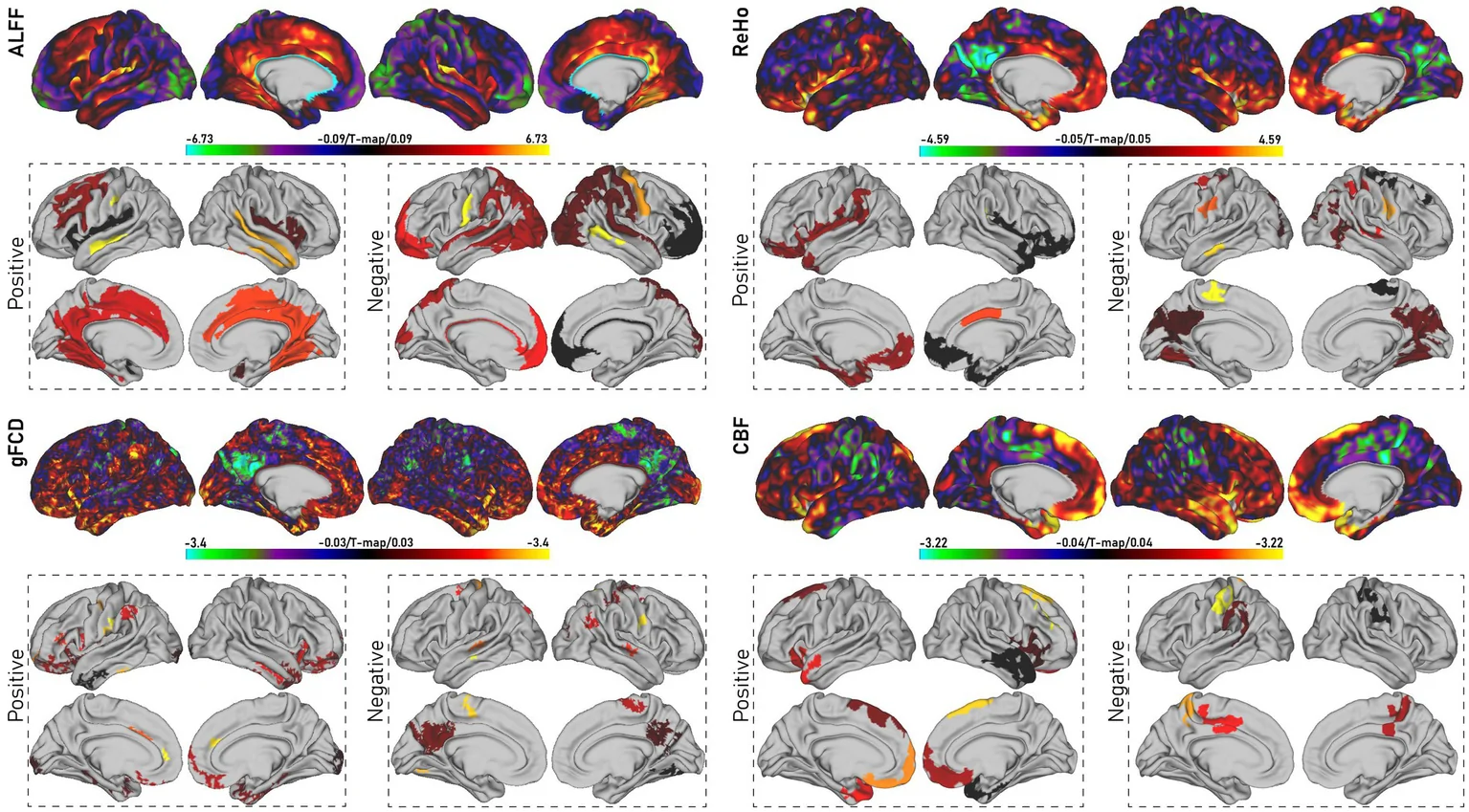
Cortical metric analyses between PD and Ctrl groups. Surface-based comparisons of cortical ALFF, ReHo, gFCD, and CBF between PD and Ctrl groups. The top row for each metric presents the statistical T-maps, with the accompanying color bars indicating the magnitude and direction of the T values. The lower panels show the significant cortical regions after multiple-comparison correction. Positive denotes regions with higher values in Ctrl than in PD, whereas Negative denotes regions with higher values in PD than in Ctrl. Abbreviations: ALFF, amplitude of low-frequency fluctuations; ReHo, regional homogeneity; gFCD, global functional connectivity density; CBF, cerebral blood flow.

### BOLD-CBF coupling

For each participant in PD and control groups, vertex-wise cortical ALFF, ReHo, and gFCD were coupled with the corresponding CBF using Pearson correlation analysis, with *p* < 0.05 considered to indicate significant coupling. The results showed that most patients with PD, as well as all healthy controls, retained significant coupling between ALFF, ReHo, and gFCD with CBF. However, a small subset of patients with PD showed a loss of this coupling relationship. These findings suggest that, during the progression of PD, cerebrovascular alterations may arise and lead to changes in cortical perfusion, which may in turn contribute to or exacerbate widespread cortical functional abnormalities (Figure 2A).

**Figure 2:**
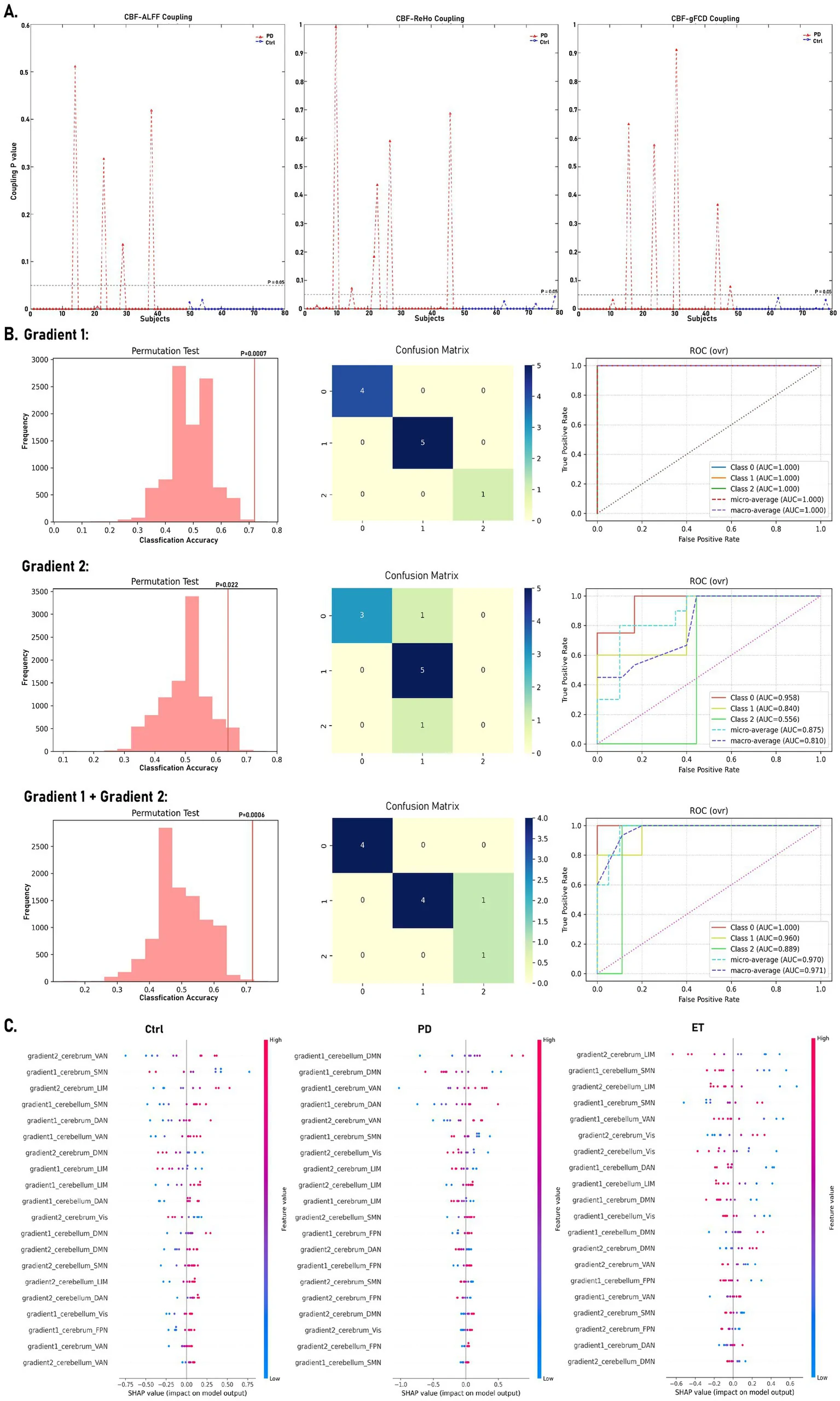
BOLD-CBF coupling and network-gradient-based classification analyses. **(A)** Subject-level BOLD-CBF coupling analyses in the Parkinson’s disease (PD) and healthy control (Ctrl) groups. Pearson correlations were calculated across cortical vertices between cerebral blood flow (CBF) and amplitude of low-frequency fluctuations (ALFF), regional homogeneity (ReHo), and global functional connectivity density (gFCD). Each vertical stem represents the correlation P value for one participant; red and blue stems denote participants with PD and HC, respectively. The horizontal dashed line indicates the nominal significance threshold of *P* = 0.05. **(B)** Classification performance of multiclass support vector machine models constructed using Gradient 1, Gradient 2, and combined Gradient 1 + Gradient 2 features. The left panels show the null distributions of classification accuracy generated by 10,000 random permutations of the diagnostic labels. The horizontal axis represents classification accuracy, the vertical axis represents permutation frequency, and the red vertical line indicates the observed classification accuracy obtained using the original labels. The empirical permutation P values are displayed above the corresponding panels. The middle panels show the confusion matrices from the best performing outer cross-validation fold using the hyperparameters selected within the corresponding training data. Rows represent the true diagnostic labels, whereas columns represent the predicted labels. The right panels show the one-versus-rest receiver operating characteristic curves and the corresponding class-specific, micro-averaged, and macro-averaged areas under the curve for the same held-out outer test fold. **(C)** SHAP summary plots showing the contributions of cortical and cerebellar gradient features to Ctrl, PD, and essential tremor (ET) classifiers. Features are ranked according to their overall importance for each classifier. Each point represents one participant; its horizontal position indicates the SHAP value, with positive and negative values increasing and decreasing the model output for the corresponding class, respectively. Point color represents the original feature value, ranging from low values in blue to high values in red. DMN, default mode network; DAN, dorsal attention network; FPN, frontoparietal control network; LIM, limbic network; SMN, somatomotor network; VAN, ventral attention network; Vis, visual network; ROC, receiver operating characteristic; AUC, area under the curve; SHAP, SHapley Additive exPlanations.

### Brain gradient analysis

Regional analyses revealed that, in PD, cortical regions showing significant alterations in Gradient 1 were predominantly distributed within DMN, DAN and SMN. In contrast, regions with significant alterations in Gradient 2 were mainly located within VN, SMN, VAN and DMN, with a smaller number of affected regions also involving DAN and FPN. By comparison, patients with ET exhibited substantially fewer cortical regions with significant alterations in either Gradient 1 or Gradient 2 relative to healthy controls than did patients with PD (Figure 3). Consistently, no significant regional differences were observed in cerebellar Gradient 1 or Gradient 2 in ET compared with controls. In contrast, significant cerebellar gradient alterations were identified in PD. Relative to controls, regions showing significant differences in cerebellar Gradient 1 were primarily located in Lobules I ~ VI, Crus I ~ II, Lobules VIIIa~VIIIb and Lobule IX, whereas alterations in cerebellar Gradient 2 were mainly distributed across Lobules I ~ V, Crus I ~ II, Lobule VIIb and Lobule VIIIa. All cerebellar parcellation labels were defined according to the SUIT cerebellar atlas (38) (Figure 4).

**Figure 3:**
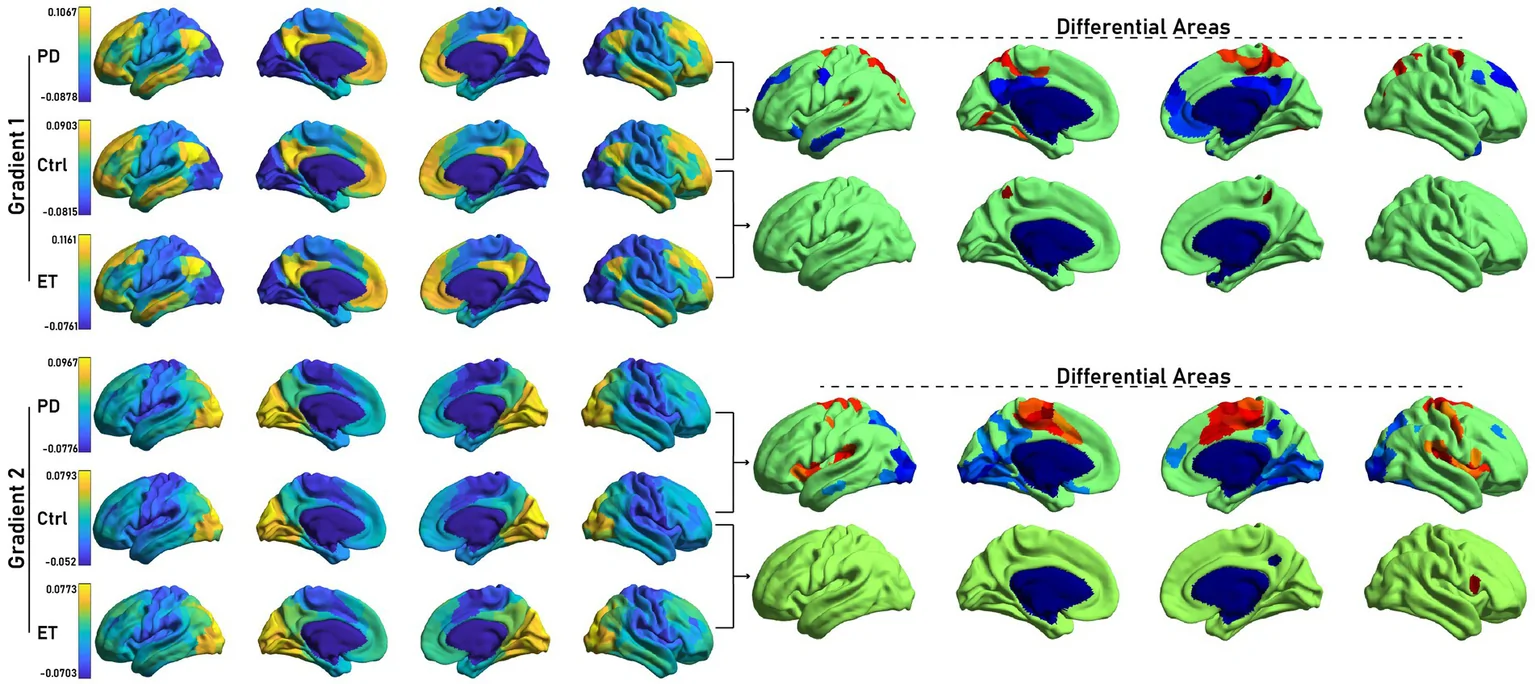
Cortical functional gradient distributions and group differences among PD, ET, and Ctrl. The left panels show group-averaged cortical maps of Gradient 1 and Gradient 2 in PD, Ctrl, and ET groups, with color bars representing gradient values. The right panels show cortical regions with significant differences between PD and Ctrl (upper row) and between ET and Ctrl (lower row). Red-to-yellow regions indicate higher gradient values in the patient group than in Ctrl, whereas blue regions indicate lower gradient values in the patient group than in Ctrl. Green regions indicate no significant group difference after multiple-comparison correction. PD, Parkinson’s disease; ET, essential tremor; Ctrl, healthy controls.

**Figure 4:**
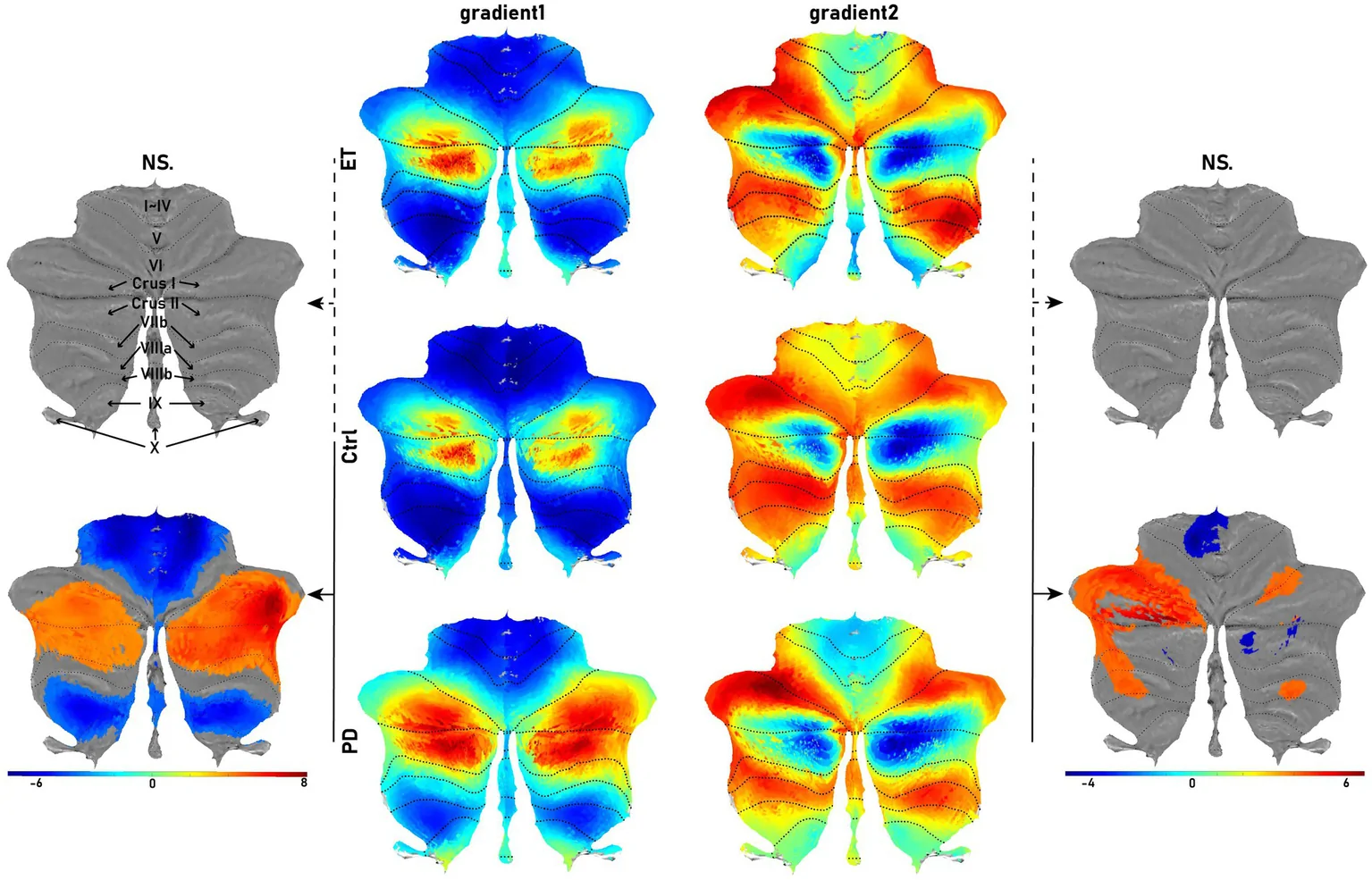
Cerebellar functional gradient distributions and group differences among PD, ET, and Ctrl. The central panels show group-averaged cerebellar Gradient 1 and Gradient 2 maps for ET, Ctrl, and PD groups, with colors representing gradient values. No significant ET-Ctrl differences were identified for either gradient after multiple-comparison correction, as shown in the upper lateral panels. The lower lateral panels show significant PD- Ctrl differences. Warm-colored regions indicate higher gradient values in PD than in Ctrl, whereas cool-colored regions indicate lower gradient values in PD than in Ctrl. The color bars represent the corresponding T values. Cerebellar lobules were identified according to the SUIT atlas. PD, Parkinson’s disease; ET, essential tremor; Ctrl, healthy controls; NS, not significant.

Kernel density distributions further showed that the group-level ranges of both cortical Gradient 1 and Gradient 2 were broader in both PD and ET than in controls, suggesting an expansion in the span of cortical functional differentiation as well as greater dispersion across modality-specific sensory systems within primary cortical regions (Figure 5A). Moreover, at the individual level, patients with PD exhibited significantly greater gradient variance and range than controls, whereas these differences were not significant in ET (Figures 5B,C). At the network level, patients with PD showed significantly lower Gradient 1 values in the visual, dorsal attention, and salience networks, but significantly higher Gradient 1 values in the frontoparietal control and default mode networks, compared with controls. For Gradient 2, patients with PD exhibited significantly lower values in SMN and VAN, whereas significantly higher values were observed in VN, DAN and DMN relative to controls (Figure 5D). In ET, network-level analyses demonstrated significantly lower Gradient 1 values in DAN and VAN, together with significantly higher Gradient 1 values in DMN, compared with controls. For Gradient 2, patients with ET showed significantly lower values in VAN, but significantly higher values in LIM and DMN relative to controls (Figure 5E).

**Figure 5:**
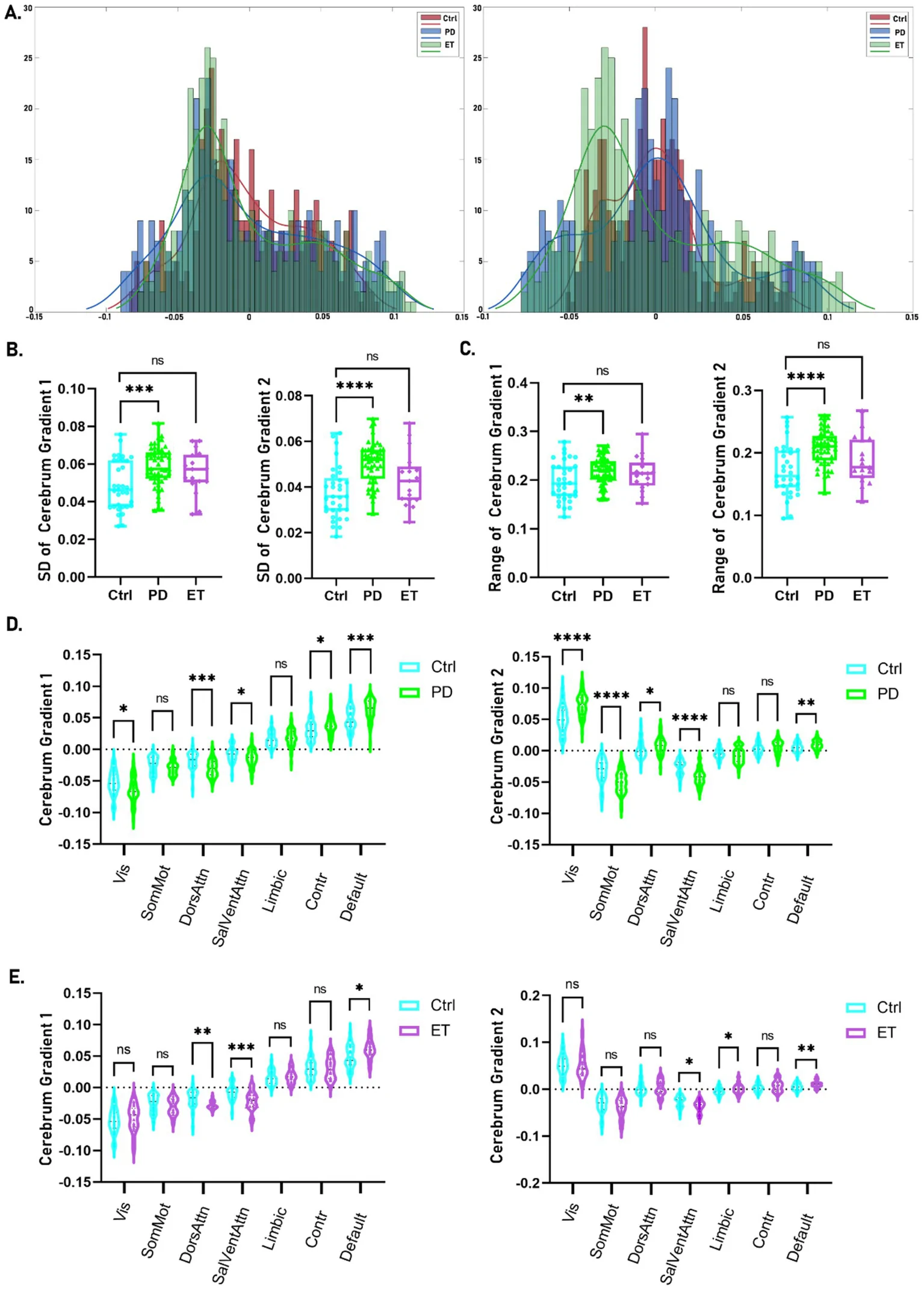
Distributional, individual-level, and network-level characteristics of cortical functional gradients in PD, ET, and Ctrl. **(A)** Histograms and kernel-density curves showing the group-level distributions of cortical Gradient 1 and Gradient 2 values in the healthy control (Ctrl), Parkinson’s disease (PD), and essential tremor (ET). **(B)** Participant-level standard deviations of cortical Gradient 1 and Gradient 2, reflecting the within-subject dispersion of gradient values across the cortex. **(C)** Participant-level ranges of cortical Gradient 1 and Gradient 2, calculated as the difference between the maximum and minimum gradient values for each participant. **(D)** Network-level comparisons of cortical Gradient 1 and Gradient 2 values between PD and HC. **(E)** Network-level comparisons of cortical Gradient 1 and Gradient 2 values between ET and HC. Boxplots and violin plots show the participant-level distributions within each group. Cortical networks were defined according to the seven-network Yeo parcellation. Statistical significance is indicated as follows: ns, not significant; ∗*p* < 0.05; ∗∗*p* < 0.01; ∗∗∗*p* < 0.001; and ∗∗∗∗*p* < 0.0001, after multiple-comparison correction as described in the Methods. Vis, visual network; SomMot, somatomotor network; DorsAttn, dorsal attention network; SalVentAttn, salience/ventral attention network; Limbic, limbic network; Cont, frontoparietal control network; Default, default mode network; PD, Parkinson’s disease; ET, essential tremor; Ctrl, healthy controls.

### Network-gradient SVM classification model

Individual-level features were extracted by aggregating cortical and cerebellar gradient values within the Yeo’s networks, and all participants from the three groups were label-encoded for subsequent classification. A multiclass support vector machine (SVM) classifier was implemented in scikit-learn using a one-versus-rest (OvR) strategy. Specifically, for each class, samples belonging to that class were treated as the positive class, whereas samples from the remaining classes were treated as the negative class, resulting in K binary SVM classifiers for K classes. During inference, each unseen sample was assigned to the class with the largest decision function value. Model performance was evaluated using a stratified 10-fold outer cross-validation scheme. Within each outer training set, network-level gradient features were standardized using parameters estimated from the corresponding outer training data, and hyperparameters were optimized using 10-fold GridSearchCV. Both a linear kernel and a radial basis function (RBF) kernel were evaluated. The hyperparameter combination yielding the highest classification accuracy within the inner cross-validation was selected for each outer fold. For visualization, the best-performing outer-fold model was used to generate the displayed confusion matrix and receiver operating characteristic (ROC) curves. Finally, model robustness was assessed using permutation testing with 10,000 iterations, with *p* < 0.05 considered to indicate statistically reliable model performance. The results showed that when Gradient 1 alone was used as the input feature set, both the macro-averaged and micro-averaged areas under the ROC curve (AUC) reached 100%, suggesting a potential risk of overfitting and optimistic performance estimation. When Gradient 2 alone was used, the model demonstrated poor discriminative performance for ET, with an AUC of only 55.6%. In contrast, when Gradients 1 and 2 from all cortical and cerebellar networks were jointly included as features, the displayed best-performing outer fold showed AUC values of 100% for healthy controls, 96.0% for PD, and 88.9% for ET (Figure 2B). SHAP analysis revealed that several gradient-derived features contributed substantially to the classification performance. In the PD classifier, features involving the cerebellar VAN, SMN and cortical DMN gradients showed the largest SHA*p* values, indicating that alterations in hierarchical functional organization across motor and higher-order cognitive networks strongly influenced model predictions. In contrast, the ET classifier was primarily driven by a smaller subset of features, suggesting relatively localized network alterations (Figure 2C).

## Discussion

The present study examined cortical and cerebellar functional gradients in PD and ET within a continuous framework of macroscale brain organization. The main implication of our findings is that PD may involve a more extensive disturbance of hierarchical organization across cortical and cerebellar systems, whereas ET appears to be associated with comparatively circumscribed alterations. Rather than reflecting isolated regional abnormalities, these changes suggest that movement disorders may differentially affect the balance between functional segregation and integration across sensory, motor, attentional, and transmodal systems. The findings therefore extend conventional region- and network-based observations by characterizing disease-related alterations along continuous axes of functional organization.

The demographic and clinical characteristics of the three groups are important for interpreting these imaging findings. Patients with PD were older than healthy controls and exhibited lower MMSE and MoCA scores, together with more pronounced depressive and anxiety symptoms. In contrast, the ET group was broadly comparable to healthy controls in age and global cognitive performance, although anxiety symptoms were more prominent. Thus, the broader gradient abnormalities observed in PD occurred in the context of greater motor, non-motor, cognitive, and affective burden. Because age and affective symptoms can influence intrinsic functional connectivity and large-scale brain organization, age, HAMD, and HAMA scores were included as covariates in the adjusted analyses. The principal pattern of cortical and cerebellar gradient abnormalities remained significant after adjustment, although several regional and network-level effects were attenuated. This suggests that the observed hierarchical reorganization was not explained solely by demographic or affective differences. Nevertheless, residual confounding cannot be completely excluded because of the cross-sectional design and the remaining imbalance in clinical characteristics.

Beyond these group-level differences, PD is clinically and biologically heterogeneous, and clinically relevant motor and non-motor phenotypes may be associated with partially distinct network abnormalities. Resting-state fMRI studies have reported altered functional connectivity in patients with freezing of gait (39), while recent work has identified altered dynamic functional connectivity and reduced higher-order information interaction in patients with PD and hyposmia (40). These observations suggest that gait dysfunction, olfactory impairment, cognitive status, affective symptoms, and other phenotypic features may contribute to interindividual variability in large-scale brain organization. The complementary measures used in the present study—ALFF, ReHo, gFCD, CBF, and functional gradients—may therefore provide a useful framework for future phenotype-stratified investigations. However, our cohort was not prospectively stratified according to freezing-of-gait status, hyposmia, or established motor subtypes. Accordingly, the current findings should be interpreted as group-level patterns of functional and hierarchical reorganization rather than phenotype-specific imaging signatures, and the present data cannot establish whether these measures identify distinct PD subgroups or symptom-specific biomarkers.

The conventional cortical metrics and functional-gradient analyses captured related but distinct aspects of brain function. ALFF reflects the magnitude of spontaneous low-frequency activity, ReHo measures local temporal synchronization, and gFCD quantifies the number of strong whole-cortex functional connections. These measures were included to provide complementary information regarding PD-related cortical dysfunction rather than as prerequisites for gradient construction or as input features for classification. By contrast, functional gradients were derived directly from FC matrices and described continuous axes of macroscale hierarchical organization. The coexistence of reduced and increased conventional functional metrics across sensory-motor and association systems is consistent with previous evidence of widespread network remodeling in PD (9, 41). Such a pattern may reflect the simultaneous presence of local dysfunction, maladaptive reorganization, and compensatory recruitment. From a gradient perspective, these regional abnormalities may collectively contribute to changes in the broader organizational axes that coordinate specialized and integrative processing.

The ET cohort was included primarily to explore whether gradient-based measures could distinguish PD from another movement disorder with overlapping tremor manifestations. Additional ET–HC and PD–ET comparisons of ALFF, ReHo, and gFCD were therefore considered exploratory. Given the relatively small ET sample, limited or nonsignificant findings in these conventional metrics should not be interpreted as evidence that cortical function is preserved in ET. Rather, they may reflect reduced statistical power and the possibility that ET-related alterations are more subtle, spatially heterogeneous, or preferentially expressed through network-level organization rather than through marked regional changes.

The principal cortical gradient is generally interpreted as an organizational axis extending from primary sensory-motor systems to transmodal association cortex. Alteration of this axis in PD may therefore reflect an imbalance between externally oriented sensorimotor processing and higher-order integrative function. The broader gradient range and greater individual-level dispersion observed in PD may indicate destabilization of the normal hierarchical relationships among large-scale functional systems. Such expansion does not necessarily imply uniformly greater functional differentiation; instead, it may reflect abnormal polarization, inconsistent positioning of functional systems, or reduced stability of their relative organization across individuals. These interpretations are consistent with the view that PD affects not only basal ganglia–motor circuits but also distributed cortical systems involved in attention, executive control, and internally directed cognition (23, 24).

By comparison, ET was associated with more restricted cortical gradient alterations and did not show the same degree of individual-level dispersion. This difference may reflect distinct pathophysiological mechanisms. PD is characterized by progressive degeneration involving multiple neurotransmitter systems and distributed cortico-subcortical networks, whereas ET is more strongly linked to abnormalities within the cerebello-thalamo-cortical system. The comparatively limited gradient changes in ET may therefore represent selective disruption of sensory-attentional and motor-regulatory processes rather than widespread alteration of the principal cortical hierarchy. However, the smaller ET sample and clinical heterogeneity of the disorder require this interpretation to remain provisional.

The cerebellar findings further support a distinction between the two disorders. In PD, gradient abnormalities involved both anterior motor-related lobules and posterior cerebellar territories, including Crus I–II and posterior lobules that have been associated with cognitive and affective networks (42, 43). This spatial distribution is anatomically compatible with the growing recognition that cerebellar involvement in PD extends beyond motor coordination. Nevertheless, the present data do not establish that these gradient abnormalities directly underlie cognitive or affective impairment. MMSE and MoCA provide measures of global cognitive performance but do not comprehensively assess domain-specific cerebellar cognitive or affective functions. Accordingly, the cerebellar findings should be interpreted as evidence of altered organization in regions anatomically connected to cognitive-affective systems rather than as direct proof of impaired cognitive-affective processing. Future studies incorporating detailed executive, visuospatial, language, and affective assessments will be required to test these associations. No significant cerebellar gradient differences were detected between ET and healthy controls. This finding does not necessarily indicate an absence of cerebellar dysfunction in ET. Previous studies have implicated the cerebellum and cerebello-thalamo-cortical circuitry in tremor generation, but such abnormalities may be expressed through focal connectivity changes, dynamic interactions, or task-dependent modulation rather than through large-scale shifts in the first two cerebellar gradients. In addition, the relatively small ET sample may have limited sensitivity to subtle or heterogeneous effects.

The conventional metrics and BOLD-CBF coupling analyses were intended to provide physiological context for the FC-derived gradient findings. The coupling results suggested interindividual heterogeneity among patients with PD, potentially reflecting differences in the relationship between spontaneous neuronal activity and cerebral perfusion. However, these observations should be interpreted cautiously. A nonsignificant within-subject correlation does not by itself establish a loss of coupling, and coupling change should ideally be quantified relative to a reference distribution and reported together with correlation coefficients and clinical associations. Therefore, the current findings should be regarded as preliminary evidence of possible neurovascular heterogeneity rather than definitive proof of impaired coupling (44). Because the ASL data from ET group did not meet the predefined quality-control criteria, perfusion and coupling analyses were restricted to PD and HC groups, preventing assessment of whether similar alterations occur in ET.

The classification analyses provide preliminary evidence that functional-gradient features contain information relevant to distinguishing PD, ET, and healthy controls (23, 45). Gradient 1 appeared to carry particularly strong discriminative information, consistent with its role in representing the principal axis of cortical organization. However, near-perfect classification in a modest and imbalanced dataset should not be interpreted as definitive evidence of complete diagnostic separability. In the current implementation, the displayed confusion matrices and ROC curves represent the best-performing outer cross-validation fold and therefore may provide an optimistic illustration of performance. More generally, high performance in a small dataset may reflect overfitting, optimistic bias, or potential data leakage, which are mechanistically distinct concerns. The combined Gradient 1 and Gradient 2 model should therefore be interpreted as incorporating complementary dimensions of hierarchical organization rather than as automatically correcting these limitations. Permutation testing indicated that the observed classification accuracy exceeded that expected under randomly permuted labels within the present dataset, but this does not establish generalizability to independent clinical populations. Accordingly, the classification analysis should be considered exploratory and requires confirmation using larger, balanced, and externally validated cohorts.

The SHAP analysis provided an exploratory description of how individual cortical and cerebellar features contributed to model predictions. The broader distribution of influential features in the PD classifier is compatible with the more extensive network-level abnormalities observed in PD, whereas the ET classifier appeared to rely on a narrower set of features (46, 47). Nevertheless, SHAP values are conditional on the validity and stability of the underlying classifier and should not be interpreted as causal evidence or as proof of disease-specific involvement of particular networks. The contribution of Gradient 2-derived features to the combined model, despite the relatively limited performance of the Gradient 2-only model, is not necessarily contradictory. The Gradient 2-only model evaluates the independent discriminatory capacity of Gradient 2, whereas SHAP values in the combined model reflect the conditional contribution of individual features in the presence of Gradient 1 and correlated network information. These attribution patterns require confirmation in larger and independent cohorts (48, 49).

The present gradient framework characterizes relatively stable axes of macroscale functional organization and does not fully capture temporal brain-state transitions or mechanistic interactions among neural systems. Future studies may therefore complement gradient analysis with dynamic functional connectivity, network modeling, information-theoretic analyses, and biophysically informed modeling. Dynamic and information-based approaches may be particularly relevant to PD heterogeneity, as illustrated by recent observations of altered dynamic connectivity and higher-order information interaction in PD with hyposmia (40). Longitudinal and interventional designs may also help test the therapeutic modifiability and functional relevance of the hierarchical abnormalities identified here. For example, comparison of gradient organization before and after dopaminergic treatment or neuromodulation, including deep brain stimulation, could determine whether changes in cortical-cerebellar hierarchy parallel clinical improvement. Previous evidence that dopaminergic medication can modulate functional gradients in PD (25) supports the sensitivity of macroscale hierarchy to intervention, although causal inferences will require dedicated longitudinal or experimental studies.

Several limitations should be acknowledged. First, the sample size was modest and imbalanced, particularly for the ET group, reducing statistical power and increasing uncertainty in class-specific performance estimates. Second, the cross-sectional design precludes causal inference regarding disease progression and hierarchical reorganization. Third, only the first two gradients were examined; higher-order gradients may capture additional aspects of functional organization. Fourth, although major demographic and affective variables were included as covariates, residual confounding related to age, cognition, medication history, disease severity, or other clinical factors may remain. Fifth, PD participants were analyzed as a single group despite heterogeneity in motor phenotype, gait dysfunction, olfactory status, cognitive profile, disease duration, and severity; the present sample was not designed or sufficiently powered for robust phenotype-specific subgroup analyses. Sixth, the absence of usable ET perfusion data prevented complete three-group comparisons of CBF and BOLD-CBF coupling. Seventh, the classification and SHAP analyses remain exploratory because of the modest and imbalanced sample, the possibility of optimistic performance estimates, and the lack of independent external validation. Finally, more detailed domain-specific cognitive and affective assessments are required to determine the clinical relevance of gradient abnormalities in cerebellar association territories.

In conclusion, the present findings suggest that PD and ET may be characterized by different patterns of macroscale functional organization. PD was associated with more extensive alteration of cortical and cerebellar hierarchy, whereas ET showed comparatively restricted changes. Functional-gradient analysis therefore provides a complementary framework for understanding distributed network dysfunction in movement disorders and may offer candidate imaging features for future studies of PD-related hierarchical reorganization and PD-ET differentiation. However, their clinical and diagnostic utility, particularly in newly diagnosed or early-stage disease, should be established through larger, longitudinal, and externally validated studies incorporating multimodal imaging and comprehensive clinical assessment.

## Conclusion

Compared with ET, PD exhibited more widespread and pronounced reorganization of whole-brain functional hierarchy. The abnormalities observed in PD were mainly characterized by hierarchical imbalance between sensorimotor networks and higher-order cognitive networks. In contrast, functional gradient alterations in ET were relatively limited, with the overall hierarchical network architecture remaining largely preserved. PD may involve the combined contribution of functional abnormalities, cerebral perfusion disturbances, and possible alterations in neurovascular coupling. These findings suggest that functional gradients may provide candidate imaging features for characterizing PD-related network reorganization and for exploratory differentiation between established PD and ET. Their diagnostic utility, particularly for early-stage disease, requires validation in larger and independent cohorts.

## Data Availability

The original contributions presented in the study are included in the article/Supplementary material, further inquiries can be directed to the corresponding author/s.
